# Proteomic Analysis of Porcine-Derived Collagen Membrane and Matrix

**DOI:** 10.3390/ma13225187

**Published:** 2020-11-17

**Authors:** Jung-Seok Lee, Goran Mitulović, Layla Panahipour, Reinhard Gruber

**Affiliations:** 1 Department of Oral Biology, School of Dentistry, Medical University of Vienna, 1090 Vienna, Austria; cooldds@yuhs.ac (J.-S.L.); layla.panahipour@meduniwien.ac.at (L.P.); 2 Department of Periodontology, Research Institute for Periodontal Regeneration, College of Dentistry, Yonsei University, Seoul 03722, Korea; 3 Proteomics Core Facility, Clinical Institute of Laboratory Medicine, Medical University of Vienna, 1090 Vienna, Austria; goran.mitulovic@meduniwien.ac.at; 4 Department of Periodontology, School of Dental Medicine, University of Bern, 3010 Bern, Switzerland

**Keywords:** collagen membrane, collagen matrix, proteomics, mass spectrometric detection, dentistry

## Abstract

Collagen membranes and matrices being widely used in guided bone regeneration and soft tissue augmentation have characteristic properties based on their composition. The respective proteomic signatures have not been identified. Here, we performed a high-resolution shotgun proteomic analysis on two porcine collagen-based biomaterials designed for guided bone regeneration and soft tissue augmentation. Three lots each of a porcine-derived collagen membrane and a matrix derived from peritoneum and/or skin were digested and separated by nano-reverse-phase high-performance liquid chromatography. The peptides were subjected to mass spectrometric detection and analysis. A total of 37 proteins identified by two peptides were present in all collagen membranes and matrices, with 11 and 16 proteins being exclusively present in the membrane and matrix, respectively. The common extracellular matrix proteins include fibrillar collagens (COL1A1, COL1A2, COL2A1, COL3A1, COL5A1, COL5A2, COL5A3, COL11A2), non-fibrillar collagens (COL4A2, COL6A1, COL6A2, COL6A3, COL7A1, COL16A1, COL22A1), and leucine-rich repeat proteoglycans (DCN, LUM, BGN, PRELP, OGN). The structural proteins vimentin, actin-based microfilaments (ACTB), annexins (ANXA1, ANXA5), tubulins (TUBA1B, TUBB), and histones (H2A, H2B, H4) were also identified. Examples of membrane-only proteins are COL12A1 and COL14A1, and, of matrix only proteins, elastin (ELN). The proteomic signature thus revealed the similarities between but also some individual proteins of collagen membrane and matrix.

## 1. Introduction

Guided bone regeneration (GBR) is a technique in which a membrane covers a bony defect for space provision and excluding soft tissue ingrowth [1,2]. The outcomes of this technique have been supported by various histologic [2] and clinical findings [3]. The events in the membrane compartments have recently been addressed as major and crucial to repairs of the bone defect [4], and we are beginning to understand the GBR healing processes at the cellular and molecular level [5,6]. GBR membranes might directly enhance bone regeneration in rat tibias [4,7] and in murine calvaria defect models [8]. Moreover, the cells infiltrating the collagen membranes were starting to be characterized in rat [9] and mouse subcutaneous (s.c.) implantation models [10]. It is likely that the collagen membrane exerts a direct impact on the infiltrating and adjacent cells [11], and a series of in vitro studies revealed cellular responses to collagen membranes [8,12]. Notwithstanding the pertinent interests in this issue, there is limited insight into the proteomic signature of collagen membranes.

Collagen membranes are derived from xenogeneic sources, including porcine tissues, following a chemical purification process to remove fat and other unwanted tissue components. Most of these tissue sources have a specific type of collagen, but mainly collagen type 1 (COL1), as a structural protein in the extracellular matrix (ECM) of dermal tissue, peritoneal tissue, ligament, and bone [13,14]. For example, antibodies displayed an immunoreactivity to COL1, a weaker immunoreactivity to COL3, and a faint immune reaction to collagen COL4 and COL6 in a porcine peritoneal membrane [15]. Consistently, the composition of the extracellular matrix of the peritoneum revealed COL1 and COL4 [14].

Collagen matrices being used for soft tissue augmentation [16] can be derived from a combination of porcine peritoneal membrane and a spongy part prepared from purified skin [10]. Skin tissue with COL1, being the most abundant source of collagen, includes many other types of fibrillar as well as non-fibrillar collagens. While COL1, COL3, and COL6 are abundantly expressed by dermal fibroblasts [17,18], COL4 and COL7 are typically present in the basement membrane of the epidermis, indicating interaction between fibroblasts and keratinocytes [18,19]. COL14 showed deposition throughout the dermis with strong subepidermal signals [20].

Collagens in the extracellular matrix (ECM) are linked to small leucine-rich repeat proteoglycan (SLRP), including decorin (DCN), biglycan (BGN), osteoglycin (OGN), lumican (LUM), and prolargin (PRELP) found in skin [21]. SLRP have a broad spectrum of functions [22], including the binding of TGF-β [23]. Peritoneal mesothelial cells may be the principal source of SLRPs in the peritoneum [24]. Another major component of the extracellular matrix is elastin (ELN), which is required for the formation of elastic fibers. ELN controls the elastic properties of connective tissues and presumably also of collagen-derived biomaterials. The presence of SLRP and ELN in collagen membrane and matrix has not yet been confirmed.

Highly sensitive mass spectrometers and improved separation methods have enabled analysis of minute sample amounts of proteins from a wider area of biological materials, such as plasma, serum, urine, bone, and saliva, just to name a few [25,26,27,28,29]. Furthermore, the use of proteomics enables the nature of interactions between biomaterials and biological systems to be determined, e.g., for tissue-biomaterials or cell-biomaterials [30]. The surface proteomic signature of biomaterials interplaying with surrounding tissue/cells provides foundation for understanding specific tissue responses and feedback to mimic the surface chemistry of natural percutaneous tissues [31]. Moreover, the proteomics of decellularized pancreatic ECM [32], dentin-based scaffold [33], or decellularized livers [34] reveal the pivotal role of ECM, such as various types of collagens in tissue regeneration. For a better understanding of the biomaterials used in GBR and soft tissue augmentation, we have performed a proteomic analysis on two commercially available collagenous biomaterials with the overall goal to identify the spectrum of collagens and other proteins of the extracellular matrix.

## 2. Materials and Methods

### 2.1. Preparing Collagen Membranes for Analysis

Three lots of porcine peritoneum-derived collagen membrane (Bio-Gide^®^, Geistlich Pharma AG, Wolhusen, Switzerland) and three lots of collagen matrix (Mucograft^®^, Geistlich Pharma AG, Wolhusen, Switzerland) derived from porcine peritoneum and skin without cross-linking [35] were prepared for proteomic analysis. Standardized fragments (1 cm × 1 cm) of the biomaterial were cut and submerged in a solution of 1 mL 1% Rapigest (Waters, Milford, MA, USA) in 50 mM triethylammonium bicarbonate (TEAB, Sigma Aldrich, St. Louis, MO, USA) for dissolving proteins eventually bound to the membrane. The membranes and matrices were sonicated for 15 min and centrifuged for 5 min at 4 °C and 10,000 rpm. The extracted proteins were digested as previously described [27]. Briefly, the protein concentration was determined using the nano spectrophotometer (DS-11 FX, DeNovix, Wilmington, NC, USA), and the proteins were reduced using 5 mM Dithiothreitol (DTT, Sigma-Aldrich, St. Louis, MO, USA) for 30 min at 60 °C and alkylated for 30 min using 15 mM Iodoacetamide (IAA, Sigma-Aldrich, St. Louis, MO, USA) in the dark. Finally, porcine trypsin (Promega, Madison, WI, USA) was added in a ratio of 1:50 (*w*/*w*). After 16 h of incubation at 37 °C, aliquots of 20 µL were prepared and stored in 0.5 mL protein low-bind vials (Eppendorf, Hamburg, Germany) at −20 °C until injection on the next day. The insoluble part was treated in the same manner as described for the extracted proteins by submerging the material in the respective solution during the sample preparation procedure, with the exception that the protein concentration was not assessed.

### 2.2. Peptide Separation

Following injection into the trapping column (µPAC trapping column, Pharma Fluidics, Gent, Belgium), the peptides were separated by nano-reverse-phase (µPAC, 200 cm separation column, Pharma Fluidics, Gent, Belgium) using an UltiMate3000 nano rapid separation liquid chromatography HPLC (Thermo Fisher, Germering, Germany) separation system. Both, the trap- and separation columns were operated at 50 °C, and the UV peptide detection at 214 nm served as quality control for the HPLC separation. The samples were loaded onto the trap column using a loading solvent of 2% acetonitrile (ACN, VWR, Vienna, Austria) in an aqueous mix of 0.1% trifluoroacetic acid (TFA)/0.01% heptafluorobutyric acid (HFBA), both purchased from Sigma-Aldrich, Vienna, Austria, at 30 μL/min and precooled to 3 °C [36]. Nano separation was performed in gradient mode at 600 nL/min. A user defined injection program was used for sample injection and additional injector and trap column wash. Every sample injection was followed by two blank runs with injections of 2,2,2-trifluoroethanol (Alfa-Aeser, Vienna, Austria) for the removal of possible sample remains in the injector or on the trap column and for the prevention of carryover in the separation system. In order to perform the label-free quantitation (LFQ), equimolar amounts of peptides were injected.

### 2.3. Mass Spectrometry (MS) Analysis

Prior to mass spectrometric detection and analysis, the peptides were detected using UV at 214 nm in a 3 nL cell. Mass spectrometric detection and tandem mass spectrometry, also known as MS/MS analysis, were performed using the Q-Exactive Plus Orbitrap BioPharma mass spectrometer (Thermo Fisher, Bremen, Germany). Peptides were introduced into the nano electrospray source (ESI) after the UV cell, and the ionization was performed using a stainless-steel needle with a 20 µm inner diameter and a 10 µm tip. The needle voltage was set to 2.8 kV in positive mode, and the top 10 ions were selected for MS/MS analysis (fragmentation). The resolution was set to 70,000 for full MS scans. Ions with a single charge were excluded from the MS/MS analysis, and fragmented ions were excluded for 60 s from further fragmentation. Raw MS/MS files were analyzed using Proteome Discoverer 2.4 (Thermo Fisher Scientific, Waltham, MA, USA) and by searching the Swissprot *Sus scrofa* database (*Sus scrofa*, https://www.uniprot.org/proteomes/UP000008227 [37], version from January 2020) using the following parameters: *Sus scrofa* as the taxonomy, modifications of carbamidomethyl on C as fixed, carboxymethylation on M as variable, peptide tolerance of 10 ppm, and an MS/MS tolerance of 0.05 Da. Trypsin was selected as the enzyme used, and two missed cleavages were allowed. The false discovery rate (FDR) was set to 1%, and a decoy database search was used for estimating the FDR. Analysis was performed with Scaffold software (version 4.11.1; Proteom Software, Portland, OR, USA) to visualize and validate complex MS/MS proteomics experiments. The STRING database was used to show protein-protein interactions (string-db.org [38]).

### 2.4. Data Availability

The mass spectrometry proteomics data have been deposited with the ProteomeXchange Consortium via the PRIDE partner repository [39,40] with the dataset identifiers PXD019298 and 10.6019/PXD019298. Furthermore, Scaffold data (software version 4.11.1) are available on request.

## 3. Results

### 3.1. Proteins Present in Collagen Membrane and Matrix

**Figure 1:**
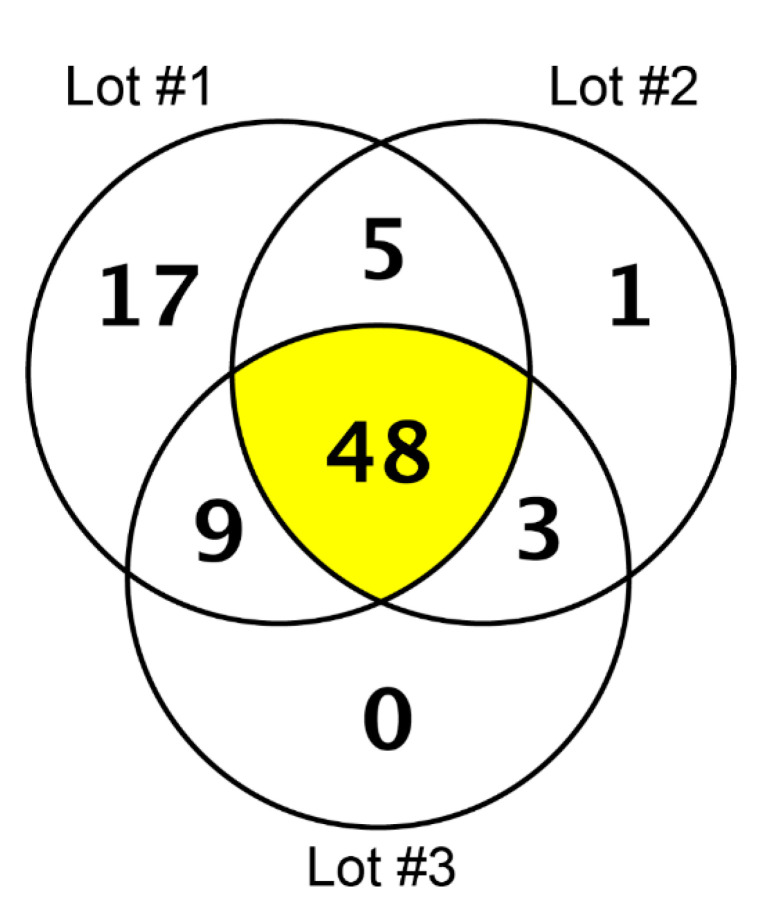
Proteins identified in three lots of the collagen membrane (Bio-Gide) by high-resolution shotgun proteomic analysis.

**Figure 2:**
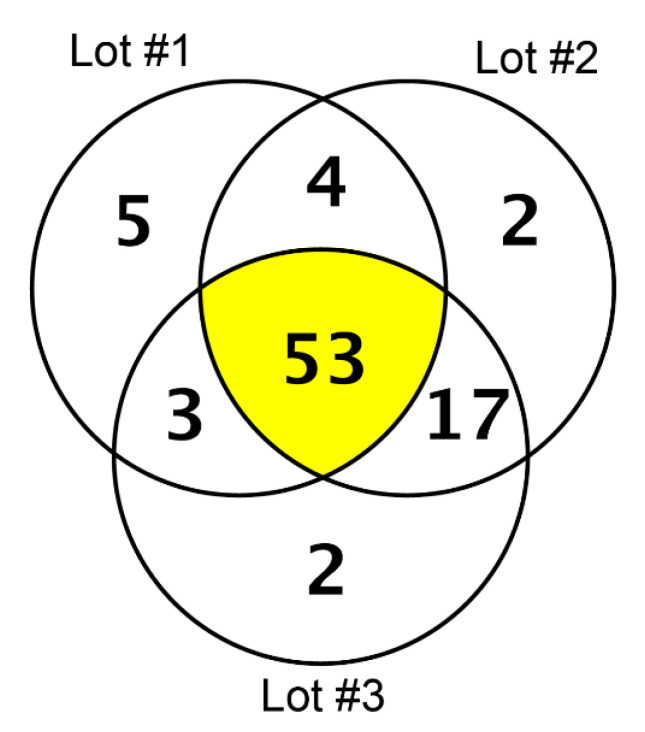
Proteins identified in three lots of the collagen matrix (Mucograft) by high-resolution shotgun proteomic analysis.

In our high-resolution shotgun proteomic analysis of three independent lots each of collagen membrane and matrix, 48 and 53 common proteins, respectively, were identified (Figure 1 and Figure 2). The vast majority of the 37 common proteins were present in both collagen membrane and matrix (Table 1). The protein spectrum of the individual lots is indicated in Supplementary Table S1.

**Figure 3:**
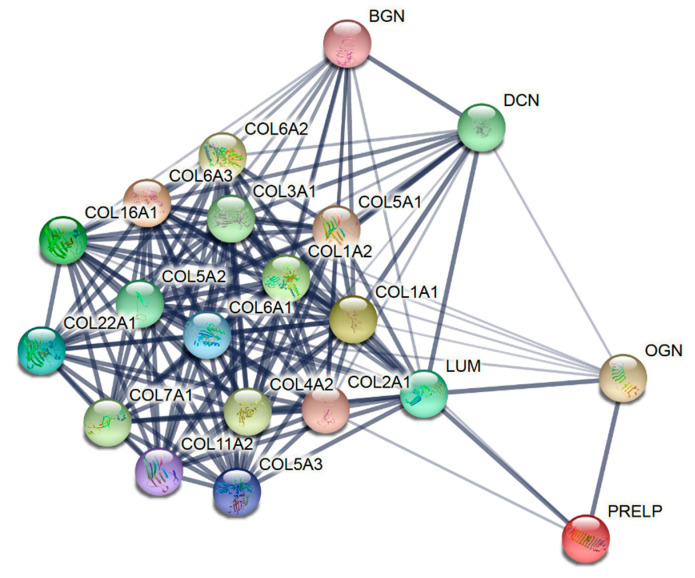
Proteins of the extracellular matrix identified in all lots of the collagen membrane and matrix by high-resolution shotgun proteomic analysis. The STRING network shows that the edges indicate both functional and physical protein associations, with line thickness representing the strength of data support.

The 37 common proteins consist of 20 members of the ECM (Figure 3). Fibrillar collagens (COL1A1, COL1A2, COL2A1, COL3A1, COL5A1, COL5A2, COL5A3, COL11A2) and non-fibrillar collagens (COL4A2, COL6A1, COL6A2, COL6A3, COL7A1, COL16A1, COL22A1) were identified in both biomaterials (Table 1). Common to the 37 proteins were also the non-collagen members of the ECM—the leucine-rich repeat proteoglycans DCN, LUM, BGN, PRELP, and OGN.

We also identified proteins that are not considered to be characteristic of the ECM, such as vimentin (VIM), a structural protein that, along with tubulin-based microtubules (TUBA1B, TUBB) and actin-based microfilaments (ACTB), comprises the cytoskeleton. In addition, annexins (ANXA1, ANXA5), histones (H2A, H2B and H4), and microfibril associated protein 4 (MFAP4) were identified as being present in three lots of membranes and three lots of matrices. Thus, the rather similar proteomic signature of the membranes and matrices includes the expected members of the ECM, as well as proteins that are linked to cellular components.

### 3.2. Proteins Present Exclusively in Collagen Membrane and Matrix

The shotgun proteomic analysis of independent lots of collagen membrane and matrix also identified 11 proteins that were exclusively present in membranes and 16 proteins in matrices (Supplement File). Among the 11 proteins are COL12A1 and COL14A1, as well as apolipoprotein A-I (APOA1), fibrinogen alpha chain (FGA), low density lipoprotein receptor-related protein 1 (LRP1), ryanodine receptor 1 (RYR1), and clathrin heavy chain (CLTC), the major protein of the polyhedral coat of coated pits and vesicles.

The 16 proteins that all three lots of the matrix have in common include elastin (ELN), elongation factor 1-alpha 1 (EEF1A1), TGF-beta-induced protein ig-h3 (TGFBI), periostin (POSTN), and asporin (ASPN). In addition, we also identified ANXA2, LRP2, histone H3, myosin-11 (MYH11), and serpin H1 (SERPINH1).

## 4. Discussion

Collagen membranes and matrices, even though being widely used in regenerative dentistry, are poorly characterized at the protein level. To serve as a scientific basis studying the impact of the protein composition on the clinical behavior of collagen membranes and matrices, we performed a proteomic analysis of a porcine peritoneum-derived collagen membrane used for GBR procedures, and a collagen matrix derived from porcine peritoneum and skin usually applied for soft tissue augmentation. Thus, there were two independent clinical indications for biomaterials. Considering that the membrane and the matrix contain peritoneum tissue and that the collagen matrix also contains collagen from skin, we expected rather similar proteomic signatures for the two biomaterials, with the collagen matrix being more heterogenous.

The main finding of this analysis was that the overall composition of both biomaterials was rather similar. Among the 37 common proteins occurring in all lots of both biomaterials, 20 were considered collagens and another five proteins were linked to the leucine-rich proteins. Thus, together, 68% of the common proteins are related to the extracellular matrix. Considering that the composition of the biomaterials reflects the composition of the extracellular matrix of the original tissue, we could confirm the presence of COL1 and COL4 in both biomaterials [14]. The proteomic signature further supports immunostainings of the collagen membrane, showing positive signals for COL1, COL3, COL4, and COL6 [15]. The present research extends these findings, allowing us to additionally identify COL2, COL5, COL7, COL9, COL11, COL16, and COL22. The broad spectrum of collagens in the matrix also reflects the complex composition of the skin with COL1, COL3, COL4, COL6, COL7, COL12, and COL14 [17,18,19,20].

Apart from the collagens, other proteins of the extracellular matrix were identified. BGN, DCN, LUM, OGN, and PRELP all belong to the SLRP and are present in skin [21], exerting a broad spectrum of functions [22], including the binding of TGF-β [23]. Peritoneal mesothelial cells may be the principal source of SLRPs in the peritoneum. Given the proposed functions of DCN and BGN, SLRPs may be involved in the control of TGF-β activity and collagen fibril formation in the peritoneum [24]. Our previous in vitro data support the binding capacity of collagen membranes for TGF-β [41,42]. Thus, the identification of various leucine rich proteins in both the membrane and the matrix reflects the composition of the original tissue and may translate into a biological activity in the biomaterial.

Collagen membranes were isolated from xenogenic tissues and subjected to defatting and alkaline treatment. Then, acid-sensitive contaminations were eliminated and the membranes dehydrated with acetone (US Patent number: US5837278A [43]). It is likely that, apart from components of the ECM, not all other proteins were removed during the processing of the xenogenic tissues. These included VIM, which, together with ACTB and ANXA1, forms clusters with ANXA5, tubulins, and MYH10 in STRING analysis. In addition, histone (HIST1H2BD), required for packing DNA into nucleosomes, indicates the remnants of cellular proteins. These remaining proteins are presumably not of concern as they are evolutionary conserved and, clinically, no adverse reaction against the collagen membranes or matrices was reported. Only a rat subcutaneous implantation model of collagen membranes revealed a giant cell and inflammatory reactions, which resulted in thin encapsulation [9]. The underlying molecular mechanism is unclear.

In general, to produce decellularized biomaterials, removing cells and immunogenic material from the xenogenic tissue is mandatory as the immune system can recognize porcine material as foreign and produce xenoreactive antibodies [34,44]. Most, but not all, xenoreactive antibodies bind to a terminal galactose (α-gal; galactose-alpha-1,3-galactose) expressed in pigs [45]. Consequently, the decellularization protocols focus on reducing α-gal epitopes [46]. Some collagen membranes were checked for their lack of α-gal [47]. Since α-gal is not identified by proteomic analysis, the absence of α-gal in the collagen membrane and matrix should be confirmed in future studies. Xenoreactive antibodies raised against bovine proteins ANXA5, OGN, and HBB have been identified in rabbit serum [48] but not in human serum so far [49]. Thus, to our knowledge, no xenoreactive antibodies to the major proteins identified in the membrane and matrix are reported.

Collagen membranes and matrices can be distinguished by the presence of skin collagen in the matrix, while the collagen membrane contains exclusively proteins from peritoneal tissue. Our descriptive analysis reveals 11 and 16 proteins that were exclusively identified in collagen membranes and matrices, respectively. What was unexpected, though, was that the membrane, but not the matrix, contains COL12A1 and COL14A1, apart from LRP1, RYR1, and CLTC. Theoretically, all membrane proteins should be identified in the matrix; however, the presence of POST and ASPN, as well as LRP2, HBA, and SERPINH1, may originate from the presence of skin collagen in the matrix. Unfortunately, the qualitative analysis does not provide insights into the relative proportion of the processed porcine peritoneum and the skin used to prepare the matrix.

Relative quantification of the proteins comparing the membrane with the matrix, for example, has shown that the matrix has comparatively more COL6 than the membrane. It can only be speculated that it is the COL6 being abundantly expressed by skin fibroblasts [50] that distinguishes the peritoneal collagen membrane from the peritoneal-skin collagen matrix. In addition, DCN, TGFBI, and PKM are relatively higher in the matrix than in the membrane, suggesting that this is caused by the presence of skin, rather than only peritoneal components. In contrast to that, COL3A1 is relatively higher in the membrane than in the matrix. However, the observations are currently not based on a systematic and statistical analysis and may lead to wrong conclusions. A quantitative proteomic analysis to better explain the difference between the proteomic signatures of the membrane and the matrix shall be performed in a follow-up study. Moreover, the proteomic analysis does not consider the three-dimensional nature of the biomaterial supposed to affect the clinical outcome in GBR, increasing keratinized tissue or recession coverage, respectively.

By analyzing the proteome of the collagen membranes [5,6], an insight into the biological activity can be achieved. For example, the mineralization of collagen fibrils within the collagen membrane in a specific condition, such as in our previous studies [51,52], may be an exemplary event showing the direct biological role of collagen membranes. It is widely known that collagen fibers provide active sites of apatite nucleation, and this mineralization might be a passive process [53]. However, fragments of collagen degradation, called matricryptins, are bioactive fragments, with tetrastatins 1–3 and endostatin originating from COL4A4 and COL18A1 being possible factors affecting osteogenesis [54]. For example, endostatin promotes soft callus formation but inhibits callus remodeling during fracture healing [55]. Moreover, the role of SLRPs, particularly DCN, BGN, and OGN (mimecan), in the mineralization of the extracellular matrix during osteogenesis is increasingly understood [56,57,58,59]. The proteomic analysis identified a number of proteins that are functionally linked to mineralization. However, it is still unclear to which extent and if at all they affect the osteoconductive properties of the collagen membranes in vivo [51].

Even though not identified by the proteomic analysis, collagen membranes contain growth factors such as TGF-β [60]. However, and consistent with the proteomic analysis, neither the collagen membrane nor the matrix holds an intrinsic TGF-β activity based on a bioassay [61]. It is more likely that locally released growth factors, such as TGF-β, adsorb to the collagen and SLRPs, thus changing the biological behavior in vivo. Proteomics may thus allow the identification of proteins, including growth factors adsorbed to the collagen membranes and matrices upon soaking in human serum. Nevertheless, the descriptive nature of the methods and the facts that the biomaterials are processed by purification and sterilization, and that thus the proteomic signature cannot be directly translated into a biological activity are limitations of the current study. Thus, the proteomic signature should be considered as one piece of a mosaic to better understand the properties of the biomaterials, and further experiments involving a larger number of different lots will be performed in order to gain more information.

## Figures and Tables

**Table 1:** The 37 proteins identified in all lots of collagen membranes and matrices.

Gene ID	Protein Name	Gene Symbol
Q6QAQ1	Actin, cytoplasmic 1	ACTB
F1SJB5	Annexin	ANXA1
A0A287A531	Annexin	ANXA5
F1S2B6	Biglycan	BGN
F1RYI8	Collagen alpha-1(III) chain precursor	COL3A1
F1S021	Collagen alpha-1(V) chain precursor	COL5A1
I3L781	Collagen alpha-2(I) chain precursor	COL1A2
A0A287BF88	Collagen alpha-2(V) chain precursor	COL5A2
F1S3G7	Collagen alpha-3(V) chain precursor	COL5A3
A0A287A1S6	Collagen type I alpha 1 chain	COL1A1
A0A286ZWS8	Collagen type II alpha 1 chain	COL2A1
F1RLL9	Collagen type IV alpha 2 chain	COL4A2
A0A287B5M9	Collagen type VI alpha 1 chain	COL6A1
I3LQ84	Collagen type VI alpha 2 chain	COL6A2
I3LUR7	Collagen type VI alpha 3 chain	COL6A3
F1SKM1	Collagen type VII alpha 1 chain	COL7A1
K7GME7	Collagen type XI alpha 2 chain	COL11A2
A0A286ZHY0	Collagen type XVI alpha 1 chain	COL16A1
F1RSI7	Collagen type XXII alpha 1 chain	COL22A1
Q9XSD9	Decorin	DCN
I3LC73	Fatty acid synthase	FASN
F1RII7	Hemoglobin subunit beta	HBB
A0A287B959	Histone H2A	HIST1H2AC
F2Z584	Histone H2B	HIST1H2BD
P62802	Histone H4	no symbol for pig
A0A286ZZ03	Ig-like and FN type III dom.-cont. protein 1	IGFN1
F1SQ09	Lumican precursor	LUM
A0A287AB52	Microfibril associated protein 4	MFAP4
A0A0H5ANC0	Mimecan precursor	OGN
F1SSA6	Myosin motor domain-containing protein	MYH10
F1S6B4	Prol. arg. rich end leuc. rich repeat protein	PRELP
I3LSK9	Retrotransposon Gag like 9	RTL9
A0A287BAY9	Serum albumin	ALB
A0A287B5W2	Trypsinogen precursor	LOC100302368
Q2XVP4	Tubulin alpha-1B chain	TUBA1B
Q767L7	Tubulin beta chain	TUBB
P02543	Vimentin	VIM

## Supplementary Materials

The following are available online at https://www.mdpi.com/1996-1944/13/22/5187/s1, Table S1: The protein spectrum of the individual lots.
